# Cold water swimming and upper respiratory tract infections

**DOI:** 10.1186/2046-7648-4-S1-A36

**Published:** 2015-09-14

**Authors:** Naomi Collier, Heather C Massey, Mitch Lomax, Mark Harper, Michael J Tipton

**Affiliations:** 1 Extreme Environments Laboratory, Department of Sport and Exercise Science, University of Portsmouth, Portsmouth, UK; 2 Consultant Anaesthetist, Royal Sussex County Hospital, Brighton, UK

## Introduction

It is often suggested that habitual cold water swimming (HCS) may improve resistance to infection [1], yet research into effects of HCS on the immune system has produced inconclusive results. This may be due to the wide range of protocols, from brief ice-cold dips [2] to long cold water swims [3]. Many studies measured blood and saliva markers rather than actual illness, and the clinical significance of these markers is not well established [4]. Incidence of upper respiratory tract infection (URTI), i.e. the common cold, is a useful indication of *in vivo *immune system function [5,6]. This study compared URTI susceptibility in those practising HCS with that in their non-swimming co-habiting partners. To control for any effect of swimming, those who swim in indoor heated pools and their partners were also investigated. The null hypothesis (H_0_) was that there would be no difference between swimming groups.

## Methods

50 couples were recruited to this ethically approved study, with 44 completing it: 21 cold water swimmers, 23 pool swimmers, and their non-swimming partners. Participants reported URTI symptoms using the Jackson Cold Scale [7], and their physical activity (PA), every week for 13 weeks from 1 December 2014. URTI frequency and severity were calculated per group each week. Each person's symptom days were weighted for severity and totalled. The sum of scores ≥14 was divided by the number of responses to give *URTI frequency*, and by the number of persons scoring ≥14 to give *URTI severity*. Group averages for the 13 weeks were compared between all groups (t-test for independent samples or Mann-Witney U) and correlations were sought between PA and URTI measures (Pearson's *r *or Spearman's *ρ*).

## Results

Cold swimmers had fewer colds than their partners (URTI frequency, mean (SD) 3.0 (3.2) v 5.5 (3.2), p = 0.03; percent with URTI 8.2% v 12.8%, p = 0.04). There were no statistically significant differences between cold and pool swimmers in any URTI measure. More pool partners than swimmers got an URTI (17.8% v 11.4%, p = 0.03). Cold partners had more severe colds than pool partners (URTI severity 41.6 (17.1) v 29.7 (8.2), p = 0.03). There were trends for correlations between weekly cold swim time and URTI rate (Spearman's *ρ *= 0.51, p = 0.07), and between weekly cold swim duration and URTI severity (*r *= 0.50, p = 0.09). There were no correlations between pool swimming and URTIs.

## Discussion

Although cold water swimmers had the lowest average URTI frequency and the lowest percent with an URTI, these were only statistically significant when compared to cold partners. URTI severity was highest in cold partners and very similar in the other three groups. Both groups of swimmers had fewer URTIs than their partners, but this does not imply any protective effect of swimming, and in respect of cold water swimming it appears that more may not be better. These are preliminary results and deeper analysis is continuing.

## Conclusion

There were no differences in URTI susceptibility between cold and pool swimmers (H_0 _accepted), therefore cold water swimming appears to have no protective effect. Cold swimmers had better resistance to colds than their partners, but the reason for this is not known.
